# Supplementary material for: Flow Orchestrated Regulatory Genomics Engine (FORGE): A Configurable Nextflow Pipeline for End-to-End snMultiome Analysis
Source: bioRxiv. 2026 Sep 16:2026.09.10.750690. Preprint. [Version 1] doi: 10.64898/2026.09.10.750690 (PMC13596538; doi:10.64898/2026.09.10.750690)
Supplement: Supplementary file 1 [file NIHPP2026.09.10.750690V1-supplement-1.pdf]

bioRxiv preprint doi: https://doi.org/10.64898/2026.09.10.750690; this version posted September 16, 2026. The copyright holder for this preprint (which was not certified by peer review) is the author/funder, who has granted bioRxiv a license to display the preprint in perpetuity. It is made available under a CC-BY 4.0 International license.

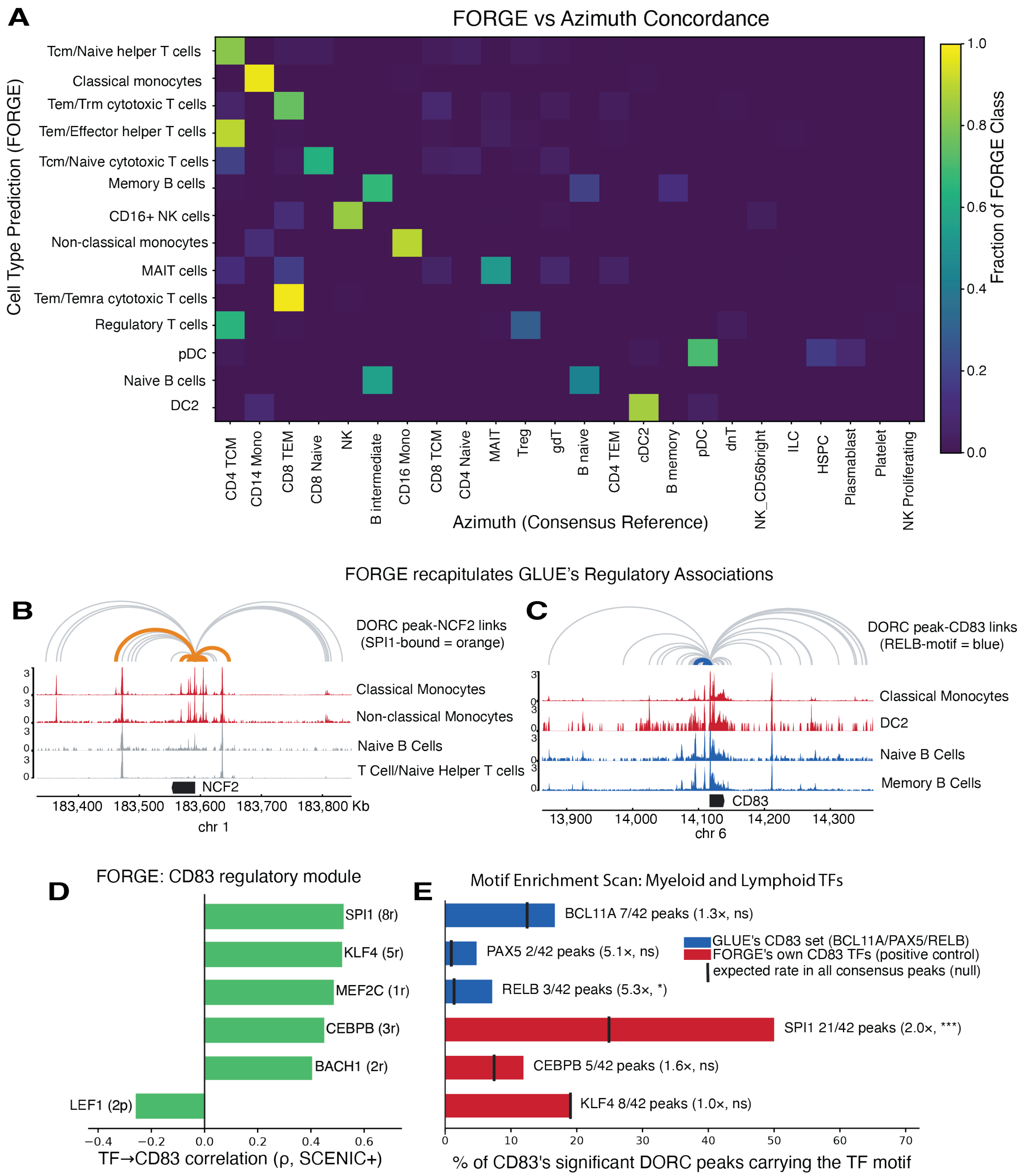

S1. FORGE produces concordant cell-type annotations to Azimuth reference projection at lineage resolution and orthogonally recapitulates regulatory associations identified by GLUE in human PBMC.

FORGE excels in biological interpretability when benchmarked against gold-standard tools for integration and generating putative cross modally justified mechanisms of action. (A) Cross-method concordance matrix of 14 FORGE broad CellTypist classes against an inferred reference of labels transferred from the multimodal PBMC reference via Seurat reference projection (FindTransferAnchors/MapQuery) wherein 24 Azimuth l2 classes are observed. The matrix is row-normalized. (ARI=0.661, AMI=0.675, 98.4% agreement

bioRxiv preprint doi: https://doi.org/10.64898/2026.09.10.750690; this version posted September 16, 2026. The copyright holder for this preprint (which was not certified by peer review) is the author/funder, who has granted bioRxiv a license to display the preprint in perpetuity. It is made available under a CC-BY 4.0 International license.

when collapsed to the lineage level). Azimuth l2 prediction score mean=0.79 and median=0.85 (11% < 0.5); per-cell mapping score mean=0.63 (30% < 0.5). 10x pbmc_granulocyte_sorted_10k n = 9,327 cells and n=7,620 paired cells. (B) FORGE recapitulates GLUE’s SPI1 to NCF2 regulatory association in monocytes. 31 significant DORC peak-gene links to NCF2; orange = 9 links carrying SPI1 SCENIC+ eRegulon region (12 SPI1 eRegulon regions target NCF2 in total). Depth-normalized ATAC pseudobulk coverage below, with B and T cells as lineage contrasts to the monocyte-biased signal. (C) scGLUE assigns lymphoid TFs whereas FORGE asserts myeloid eRegulons; additionally, FORGE corroborates possible CD83-RELB regulatory action but finds insufficient evidence for scGLUE identified TFs, BCL11A and PAX5. Of the 42 significant DORC links, blue arcs indicate 3 peaks carrying a RELB motif. Depth-normalized ATAC psuedobulk coverage in myeloid and lymphoid cells demonstrates CD83 accessibility in both lineages. (D) FORGE's CD83 regulatory module (SCENIC+). Bars give ρ, the correlation across cells between each TF's expression and CD83 expression (rho_TF2G), split at zero by sign; LEF1 is the only negatively correlated TF and is called arepressor by SCENIC+. Parentheses give the number ofdistinct regions (r) linking that TF to CD83. Each is a (TF,region, gene) triplet with three independent lines of evidence: enrichment of the TF's binding motif in the region, region accessibility vs CD83 expression correlation, and lastly correlation between TF and CD83 expression. None of GLUE's CD83 TFs (BCL11A, PAX5, RELB) reach CD83 per FORGE’s SCENIC+ implementation. (E) Matched-layer motif test. GLUE's CD83 call comes from a permissive peak-gene graph, so a more comparable FORGE layer is DORC, not eRegulons. Bars give the percentage of CD83's 42 significant DORC peaks overlapping (≥1 bp) each TF's pycisTarget cistrome. Black ticks give that TF's cistrome as a fraction of all 172,884 consensus peaks. Blue = GLUE's CD83 set, red = FORGE's own CD83 TFs (positive control). SPI1 21/42 vs 10.5 expected = 2.0× (one-sided binomial p=3.9e-4, significant after Bonferroni correction across the six TFs tested); RELB 3/42 vs 0.6 expected = 5.3× (uncorrected p=0.019, n.s. after correction); PAX5 2/42 = 5.1× and BCL11A 7/42 = 1.3×, neither significant.

bioRxiv preprint doi: https://doi.org/10.64898/2026.09.10.750690; this version posted September 16, 2026. The copyright holder for this preprint (which was not certified by peer review) is the author/funder, who has granted bioRxiv a license to display the preprint in perpetuity. It is made available under a CC-BY 4.0 International license.

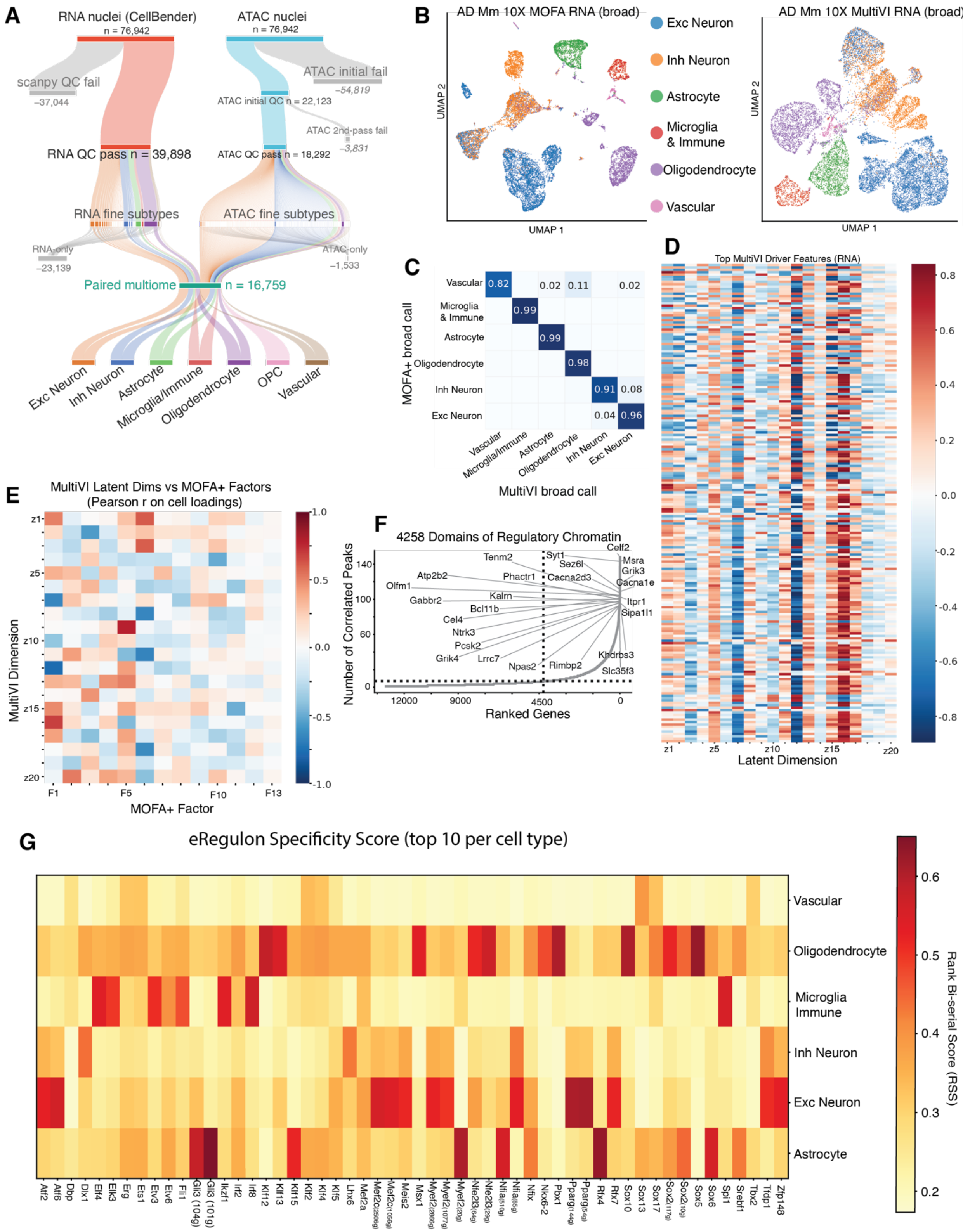

bioRxiv preprint doi: https://doi.org/10.64898/2026.09.10.750690; this version posted September 16, 2026. The copyright holder for this preprint (which was not certified by peer review) is the author/funder, who has granted bioRxiv a license to display the preprint in perpetuity. It is made available under a CC-BY 4.0 International license.

Figure S2. FORGE's global descriptive architecture scales to multiple samples in a mouse disease model. FORGE end-to-end analysis in an Alzheimer's disease mouse model, demonstrating QC outputs, integrated dimensional reduction, and gene regulatory network inference in a second species and tissue. (A) Quantitative summary of FORGE QC steps, which retain 39,898 of 76,942 CellBender-denoised RNA nuclei and 18,292 two-pass-filtered ATAC nuclei to identify 16,759 paired multiome nuclei distributed among seven broad cell types. Ribbon width is proportional to nuclei number and modality-restricted nuclei (RNA-only, 23,139; ATAC-only, 1,533) are excluded from downstream analysis. (B) UMAP visualizations of MOFA+ and MultiVI multiome latent embeddings colored by broad RNA annotation, showing that both orthogonal integrations recover the same broad tissue architecture. (C) A KNN classification task assessing agreement between broad cell type annotations within orthogonal latent multiome embeddings, with concordance ≥0.82 for all major classes. (D) Gene expression signatures associated with the top 20 MultiVI dimensions suggest varying levels of the sensitivity of specific MultiVI dimensions to changes in expression. (E) Pearson correlations between MOFA+ factor cell loadings and MultiVI latent embedding dimension cell loadings show emergent agreement among orthogonal methods without one-to-one factor correspondence. (F) 4,258 domains of regulatory chromatin (DORC) orthogonally computed via scPrinter, dominated by synaptic and neuronal loci, corroborate various eRegulon signals; the dotted line marks the correlated-peak threshold and the dashed line the resulting DORC cutoff. (G) Various transcription factors plotted by their respective eRegulon's rank bi-serial score show oligodendrocytes and excitatory neurons dominate the strongest signal for potential eRegulon-based mechanisms of action, with canonical lineage regulators observed.

bioRxiv preprint doi: https://doi.org/10.64898/2026.09.10.750690; this version posted September 16, 2026. The copyright holder for this preprint (which was not certified by peer review) is the author/funder, who has granted bioRxiv a license to display the preprint in perpetuity. It is made available under a CC-BY 4.0 International license.

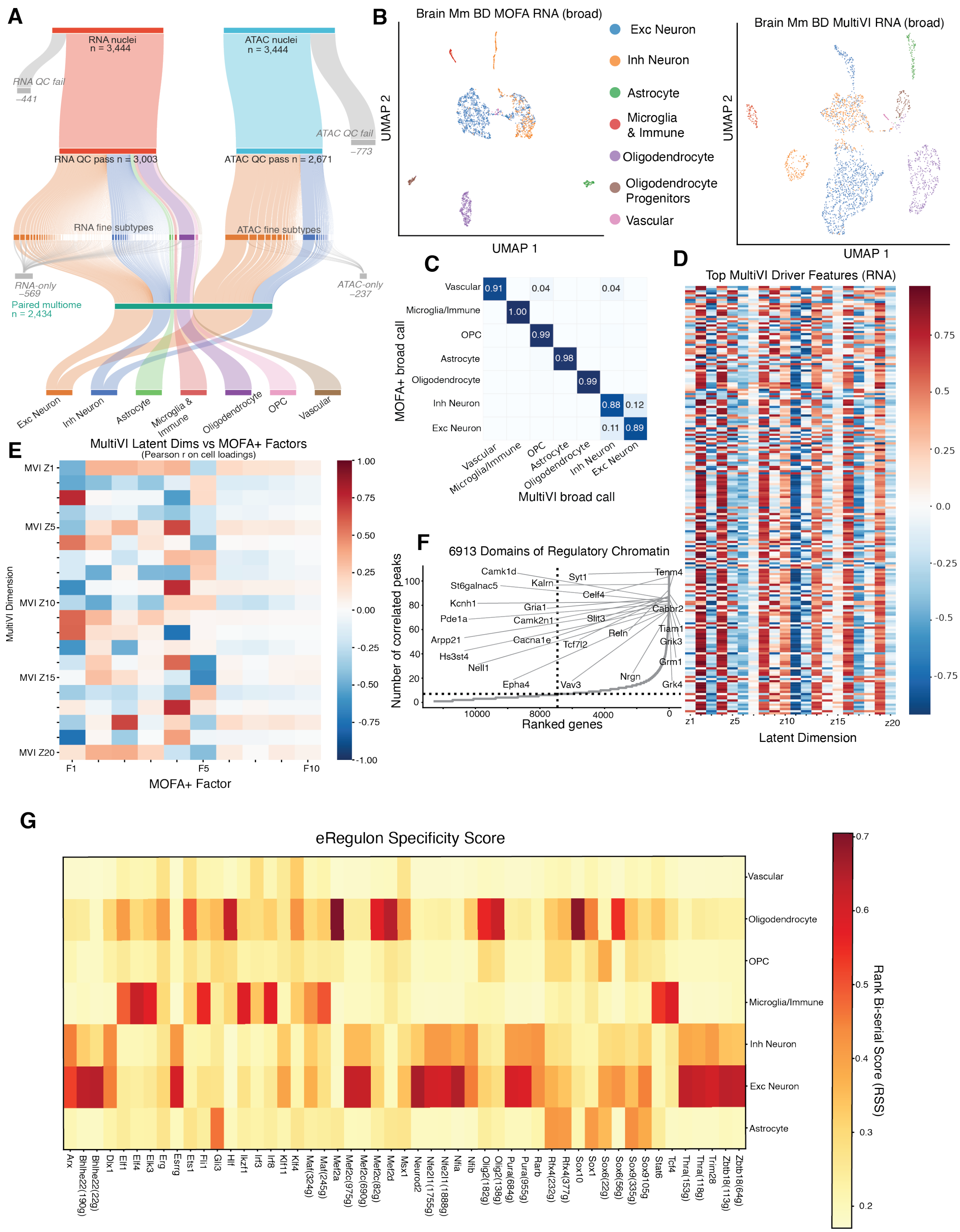

Figure S3. FORGE performs consistently across library preparation chemistries, recovering similar cell type structure and regulatory programs in a BD/Waters mouse brain multiome. FORGE end-to-end analysis in a healthy control mouse brain, demonstrating QC outputs, integrated dimensional reduction,

bioRxiv preprint doi: https://doi.org/10.64898/2026.09.10.750690; this version posted September 16, 2026. The copyright holder for this preprint (which was not certified by peer review) is the author/funder, who has granted bioRxiv a license to display the preprint in perpetuity. It is made available under a CC-BY 4.0 International license.

and gene regulatory network inference in a second platform chemistry. (A) Quantitative summary of FORGE QC steps applied to a BD/Waters mouse brain multiome (3,444 nuclei per modality), retaining 3,003 RNA and 2,671 ATAC nuclei and identifying 2,434 paired multiome nuclei distributed among seven broad cell types. Ribbon width is proportional to nuclei number and modality-restricted nuclei (RNA-only, 569; ATAC-only, 237) are excluded from downstream analysis. (B) UMAP visualizations of MOFA+ and MultiVI multiome latent embeddings colored by broad RNA annotation. (C) Excitatory-inhibitory neuron boundary subtyping errors dominate discrepancies in a KNN classification task assessing agreement between broad cell type annotations within orthogonal latent multiome embeddings, with concordance ≥0.98 for all non-neuronal classes. (D) Gene expression signatures associated with the top 20 MultiVI dimensions suggest varying levels of the sensitivity of specific MultiVI dimensions to changes in expression. (E) Pearson correlations between cell loadings within MOFA+ factors (F1-F10) and MultiVI latent embedding dimensions (z1-z20) show emergent agreement among orthogonal methods, concentrated in the leading MOFA+ factors. (F) 6,913 domains of regulatory chromatin (DORC) orthogonally computed via scPrinter corroborate various eRegulon signals; the dotted line marks the correlated-peak threshold and the dashed line the resulting DORC cutoff. (G) Various transcription factors plotted by their respective eRegulon's rank bi-serial score show oligodendrocytes and excitatory neurons dominate the strongest signal for potential eRegulon-based mechanisms of action, with canonical lineage regulators observed.

bioRxiv preprint doi: https://doi.org/10.64898/2026.09.10.750690; this version posted September 16, 2026. The copyright holder for this preprint (which was not certified by peer review) is the author/funder, who has granted bioRxiv a license to display the preprint in perpetuity. It is made available under a CC-BY 4.0 International license.

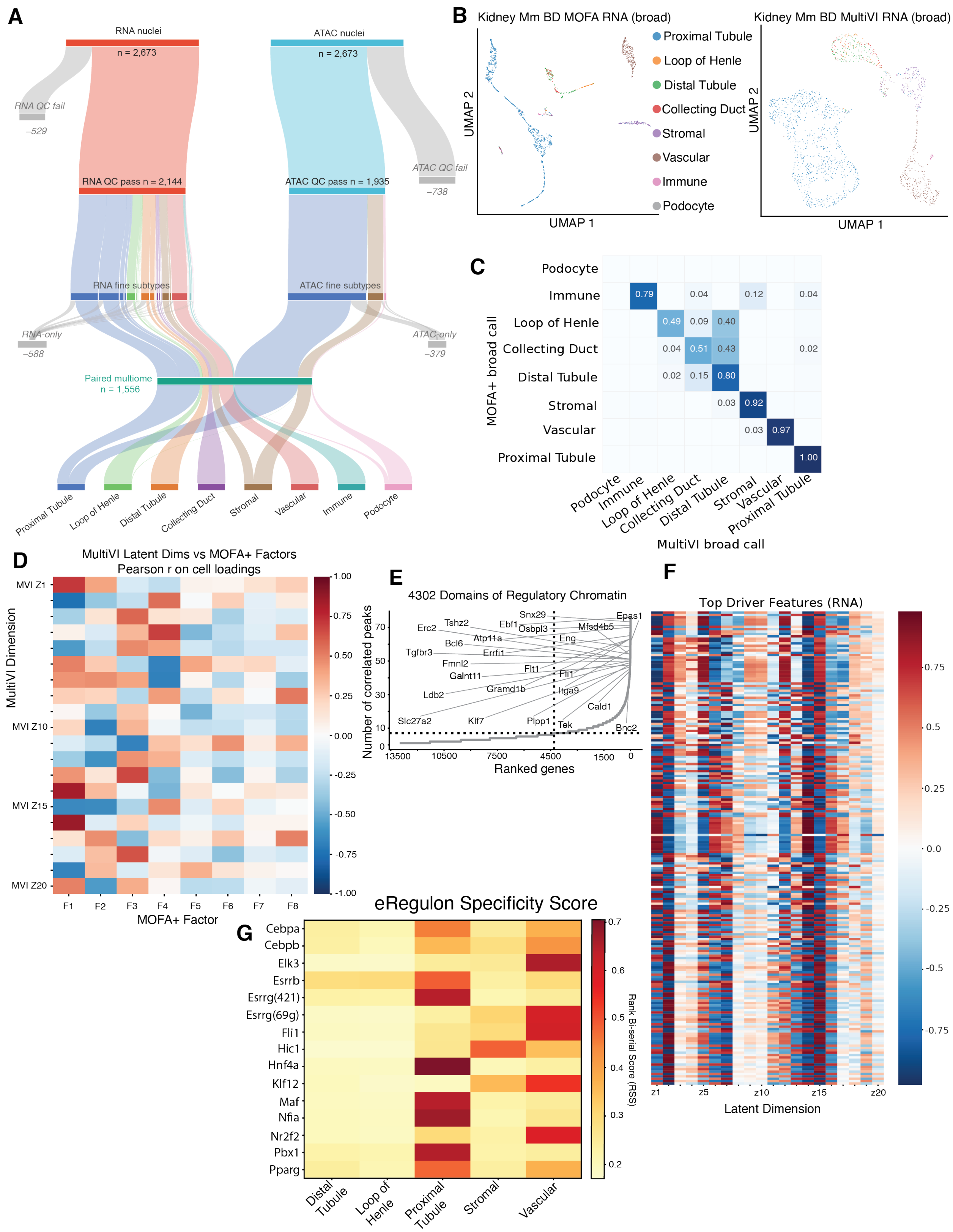

Figure S4. FORGE recovers kidney cell type structure and canonical nephron regulatory programs from a BD/Waters mouse kidney multiome.

bioRxiv preprint doi: https://doi.org/10.64898/2026.09.10.750690; this version posted September 16, 2026. The copyright holder for this preprint (which was not certified by peer review) is the author/funder, who has granted bioRxiv a license to display the preprint in perpetuity. It is made available under a CC-BY 4.0 International license.

FORGE end-to-end analysis in a healthy control mouse kidney, demonstrating QC outputs, integrated dimensional reduction, and gene regulatory network inference in a second tissue with an alternative platform chemistry. (A) Quantitative summary of FORGE QC steps applied to a BD/Waters mouse kidney multiome (2,673 nuclei per modality), retaining 2,144 RNA and 1,935 ATAC nuclei and identifying 1,556 paired multiome nuclei distributed among eight broad cell types. Ribbon width is proportional to nuclei number and modality-restricted nuclei (RNA-only, 588; ATAC-only, 379) are excluded from downstream analysis. (B) UMAP visualizations of MOFA+ and MultiVI multiome latent embeddings colored by broad RNA annotation. (C) Loop of Henle and collecting duct subtyping errors dominate discrepancies in a KNN classification task assessing agreement between broad cell type annotations within orthogonal latent multiome embeddings. Observed concordance was ≥0.92 for proximal tubule, vascular, and stromal compartments while misassignment of contiguous tubule segments was biased toward distal tubule populations. (D) Gene expression signatures associated with the top 20 MultiVI dimensions suggest varying levels of the sensitivity of specific MultiVI dimensions to changes in expression. (E) Pearson correlations between MOFA+ factor cell loadings (F1-F8) and MultiVI latent embedding dimension cell loadings (z1-z20) show emergent agreement among orthogonal methods and illuminate opportunities for exploration where anti-correlations indicate non-linear biology. (F) 4,302 domains of regulatory chromatin (DORC) (≥7 correlated peaks) orthogonally computed via scPrinter corroborate various eRegulon signals; the dotted line marks the correlated-peak threshold and the dashed line the resulting DORC cutoff. (G) Various transcription factorss plotted by their respective eRegulon's rank bi-serial score show proximal tubule and vascular compartments dominate the strongest signal for potential eRegulon-based mechanisms of action; parenthetical values indicate the number of target genes per eRegulon. Cell types with insufficient nuclei for stable eRegulon scoring (podocyte, immune, collecting duct) are not shown.

bioRxiv preprint doi: https://doi.org/10.64898/2026.09.10.750690; this version posted September 16, 2026. The copyright holder for this preprint (which was not certified by peer review) is the author/funder, who has granted bioRxiv a license to display the preprint in perpetuity. It is made available under a CC-BY 4.0 International license.

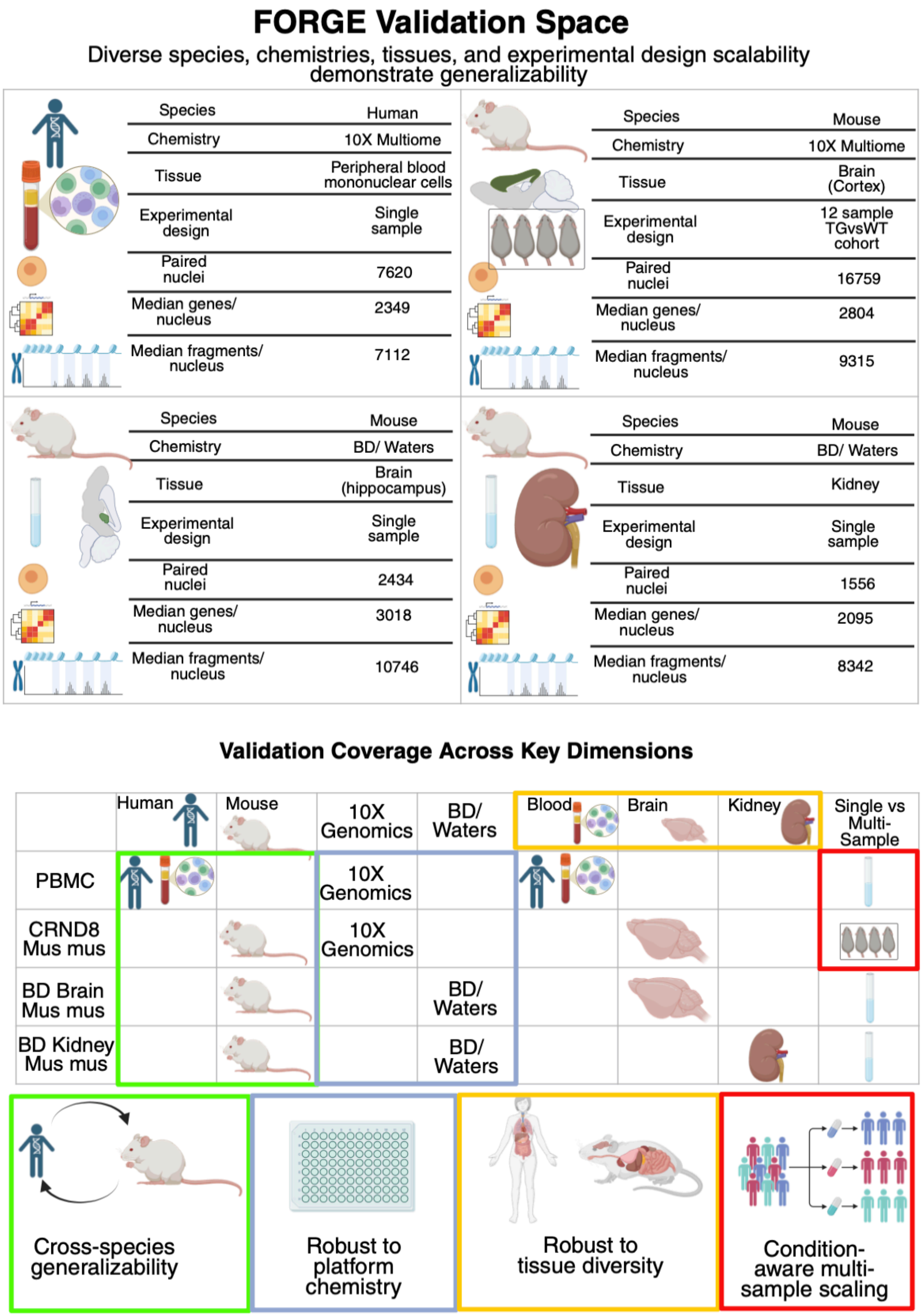

bioRxiv preprint doi: https://doi.org/10.64898/2026.09.10.750690; this version posted September 16, 2026. The copyright holder for this preprint (which was not certified by peer review) is the author/funder, who has granted bioRxiv a license to display the preprint in perpetuity. It is made available under a CC-BY 4.0 International license.

Figure S5. The FORGE validation space comprising diverse species, platform chemistries, tissues, and experimental designs demonstrates generalizeability.
Summary characteristics of the four snMultiome datasets used to validate FORGE. For each dataset, species, library chemistry, tissue of origin, experimental design, number of paired nuclei passing FORGE quality control, and median genes and ATAC fragments recovered per nucleus are reported: human PBMC (10x Multiome, single sample), mouse cortex (10x Multiome, 12-sample transgenic versus wild-type cohort), mouse hippocampus (BD/Waters, single sample), and mouse kidney (BD/Waters, single sample). Coverage matrix indicating which validation dimension each dataset addresses. Rows are datasets; columns are species, chemistry, tissue, and experimental design. Cross-species generalizability (green; human PBMC versus mouse datasets), robustness to platform chemistry (blue; 10x versus BD/Waters within mouse), robustness to tissue diversity (yellow; blood, brain, and kidney), and condition-aware multisample scaling (red; single-sample designs versus the 12-sample CRND8 cohort).

bioRxiv preprint doi: https://doi.org/10.64898/2026.09.10.750690; this version posted September 16, 2026. The copyright holder for this preprint (which was not certified by peer review) is the author/funder, who has granted bioRxiv a license to display the preprint in perpetuity. It is made available under a CC-BY 4.0 International license.

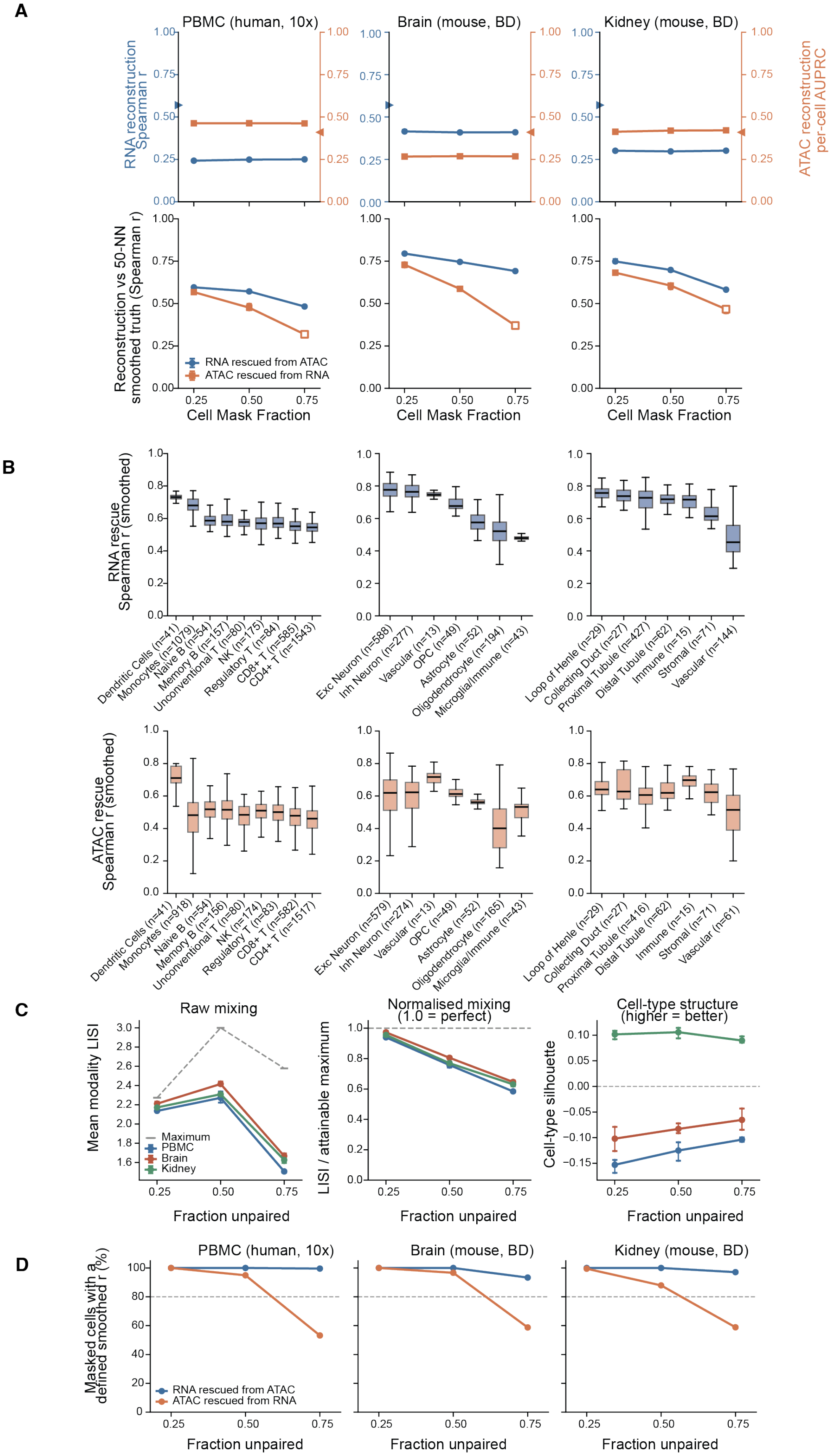

bioRxiv preprint doi: https://doi.org/10.64898/2026.09.10.750690; this version posted September 16, 2026. The copyright holder for this preprint (which was not certified by peer review) is the author/funder, who has granted bioRxiv a license to display the preprint in perpetuity. It is made available under a CC-BY 4.0 International license.

Figure S6. Cross-modal reconstruction under artificial unpairing.
Across three tissues, two species and two chemistries, FORGE's MultiVI integration reconstructs a withheld modality with fidelity that is statistically indistinguishable at 25%, 50% and 75% artificial unpairing (A) Top Row: Reconstruction of a withheld modality as a function of the fraction of cells artificially masked, for three FORGE datasets (columns). Y-axes illustrate per-cell Spearman correlation of imputed versus true expression for cells denied RNA (blue, left axis, circles) and per-cell AUPRC of imputed versus true accessibility for cells denied ATAC (orange, right axis, squares). The two axes span the same 0-1 range and neither is rescaled, though they carry different metrics whose absolute levels are not comparable; thus the panel should be read for slope within each series independently. Carets on each spine mark the corresponding MultiVI-publication benchmark. Bottom Row: both directions scored against a 50-nearest-neighbour smoothed target, which is a common scale. Points are the mean of three per-seed medians. Hollow markers flag points where the metric was undefined for >20% of masked cells (see D). (B) Reconstruction by broad cell class at 50% masking, pooled over seeds; boxes show median and IQR, whiskers 1.5 x IQR, ordered by RNA-rescue median with the ATAC row held inthe same order; n is masked cells per seed. (C) Joint-latent quality. Left: raw modality LISI against its composition-dependent attainable maximum (dashed). Middle: the same score normalised by that maximum. Right: cell-type silhouette. (D) Percentage of masked cells for which the smoothed correlation in (A, bottom row) is defined; the smoothed target is built from the masked object, so it becomes uninformative for ATAC at high unpairing. Dashed line, 80%.

bioRxiv preprint doi: https://doi.org/10.64898/2026.09.10.750690; this version posted September 16, 2026. The copyright holder for this preprint (which was not certified by peer review) is the author/funder, who has granted bioRxiv a license to display the preprint in perpetuity. It is made available under a CC-BY 4.0 International license.

Table S1. Container images and their principal packages

Image	Base image	Principal packages
scgpu_extended.sif	scverse/scvi-tools:py3.11-cu12-base	scvi-tools, CellBender (pinned commit 4334e89), CellTypist, scanpy, anndata, muon, mudata, mofapy2, Scrublet, PyTorch, NumPy, SciPy, scikit-learn, statsmodels
snapatac_extended.sif	python:3.10-slim	SnapATAC2, MACS3, scPrinter, scATAnno, harmonypy, PyTorch, CuPy, RAPIDS RMM, NumPy, SciPy, scikit-learn, statsmodels, anndata, scanpy, pyBigWig, pyGenomeTracks, logomaker, tangermeme, MOODS, pyranges, bioframe, deepTools
scenicplus.sif	python:3.11.8-slim	SCENIC+, pycisTopic, pycistarget, pySCENIC, MALLET, bedtools, graph-tool (isolated conda environment), NumPy, SciPy, pandas, statsmodels
seurat_extended.sif	rocker/r-ver:4.4.3	Seurat, SeuratObject, MAST, edgeR, limma, statmod, locfit, hdWGCNA, WGCNA, CellChat, enrichR, UCell, harmony, tidyverse, zellkonverter, schard
cicero.sif	condaforge/mambaforge	Cicero, monocle3, Gviz, rtracklayer, GenomicRanges

Table S2. Minimum cell and group sizes by analysis

Analysis	Floor	Unit
All per-cell-type fan-out (default)	max(50, 0.01N)	cells per cell type
Differential accessibility, cell-level	50	cells per condition group
Differential TF accessibility	50	cells per condition group
chromVAR permutation contrast	10 (flagged below this)	cells per group
Pseudobulk column occupancy	10	cells per (cell type, sample)
Pseudobulk replication	3	retained columns per condition
Co-expression networks	100 cells; 20 metacells	cells, then metacells per cell type
Cicero per cell type x condition	250	cells per stratum
Cell-cell communication	10	cells per sender or receiver population

bioRxiv preprint doi: https://doi.org/10.64898/2026.09.10.750690; this version posted September 16, 2026. The copyright holder for this preprint (which was not certified by peer review) is the author/funder, who has granted bioRxiv a license to display the preprint in perpetuity. It is made available under a CC-BY 4.0 International license.

Analysis	Floor	Unit
Enhancer program decomposition	200	cells per group
Coverage track export	100	cells per group

1044 **Table S3. Validation datasets**

Dataset	Species	Platform	Samples	Design	Reference build
PBMC (pbmc_granulocyte_sorted_10k)	Human	10x Chromium X	1	Single healthy donor	GRCh38, GENCODE v38, ENCODE hg38 blacklist v2
AD (TgCRND8 model)	Mouse	10x Multiome	12	Differential, TG versus WT	mm10, GENCODE vM10, mm10 blacklist v2
Brain	Mouse	BD Rhapsody multiome	1	Single control	GRCm39, GENCODE vM37
Kidney	Mouse	BD Rhapsody multiome	1	Single control	GRCm39, GENCODE vM37
